# The Oral Health Status and Treatment Needs of Pediatric Patients Living with Autism Spectrum Disorder: A Retrospective Study

**DOI:** 10.3390/dj10120224

**Published:** 2022-11-28

**Authors:** Sara Hasell, Ahmed Hussain, Keith Da Silva

**Affiliations:** College of Dentistry, University of Saskatchewan, Saskatoon, SK S7N 5A2, Canada

**Keywords:** autism spectrum disorder, pediatric dentistry, dental caries, dental restorations, permanent, general anesthesia, sedation, access to care

## Abstract

Background: The objective of this retrospective study was to assess the oral health status and treatment needs of children with ASD and to explore the differences in risk factors and oral health care status and the risk factors for treatment under GA. Methods: Dental charts of children between 6 and 14 years of age who were examined at a dental facility associated with the College of Dentistry, University of Saskatchewan between 2016 to 2019 were assessed. Children who were identified as having ASD, as well as an age- and gender-matched control group consisting of otherwise healthy children were included in the study. Results: The sample included 346 dental records, with 173 children having a diagnosis of ASD. Children diagnosed with ASD had significantly higher experience with caries (91.3% vs. 65.9%, *p* = 0.003) and severity (mean DMFT/dmft = 8.18 ± 1.62 vs. 4.93 ± 0.58 *p* = 0.007). Children with ASD were also older when visiting the dentist for the first time (age of 5.97 ± 1.18 vs. 2.79 ± 1.09, *p* = 0.02)). Children with ASD were less likely to brush once a day (66.5% vs. 88.4%, *p* = 0.02), were more likely to have bruxism (35.8% vs. 10.4%, *p* = 0.003) and were less likely to have class I occlusion (64.7% vs. 80.9%, *p* = 0.03). Findings from the logistic regression analysis revealed that children with ASD were also 2.13 times more likely to receive a referral for general anesthesia when all other variables were held constant (*p* = 0.03). Conclusions: This research demonstrates that children diagnosed with ASD may face more barriers with access to oral health care, leading to poorer outcomes and greater treatment dental needs.

## 1. Introduction

Autism Spectrum Disorder (ASD) is a lifelong neurodevelopment disorder with a magnitude of variation in individuals’ behaviours, intelligence, and abilities [1,2,3]. It is often characterized as impairments in speech, social interactions, and repetitive behaviours [4]. ASD has become a focus of research in the last 50 years, as it has transformed from a rare childhood onset syndrome to a highly advocated for and well known lifelong genetic syndrome [5]. ASD is categorized under the DSM-5 (Diagnostic and Statistical Manual of Mental Disorders) and is an umbrella term that encompasses diagnoses such as autistic disorder, Asperger’s disorder, childhood disintegrative disorder and pervasive neurodevelopmental disorder [1,3,6,7]. This spectrum of disorders is thought to be a result of both genetic and environmental components and is primarily managed through a combined program of education, behavioural therapy, and medication [4,5]. ASD affects approximately 1 in 94 Canadians and the spectrum of symptoms in ASD can range from mild to severe and must be present from early childhood [5,8,9,10]. Prevalence rates for autism are 4–5 times higher in males than females, with early indicators being recognizable around the age of 3, but diagnosis can be made as early as 15–24 months [5,11,12].

Vulnerable populations, such as those with ASD face continuous barriers in gaining timely access to oral healthcare [13,14]. These populations are often disproportionally affected by poor oral health and face heightened challenges with access to oral healthcare [9,14]. Previous studies have shown that those populations living with one or more disabilities have more missing teeth and increased prevalence of dental disease in contrast to their non-disabled counterparts [15]. However, research on the oral health status of children with autism has led to conflicting findings. Most research has shown that children with ASD have higher caries prevalence, and poorer oral hygiene in comparison to the control groups however some studies have shown children with ASD to have less dental disease as measured by the sum of decayed, missing, and filled adult or primary teeth (DMFT/dmft) index [8,12,15,16,17,18,19]. Thus, the need for more exploration into the relationship between oral health status and ASD diagnosis is needed.

ASD is a lifelong disorder that not only affects the individuals, but their families and caretakers. Individuals with ASD are often diagnosed with one or more comorbid conditions such as anxiety, depression, gastrointestinal disorders, epilepsy, attention deficit disorder and intellectual impairments [4,11,20]. The American Academy of Pediatric Dentistry (AAPD) recognizes that individuals requiring special health care needs can be at increased risk for developing a buildup of calculus, gingivitis, periodontitis, enamel hypoplasia, dental caries, oral aversion and behaviour problems, dental crowding, malocclusions, anomalies in tooth development, bruxism and tooth fracture or trauma [21]. Additionally, impairments to cognitive, intellectual, language, and social abilities combined with limited adaptive behaviour may make cooperation difficult in a dental setting, resulting in heightened fear and anxiety associated with dental treatment. As such, children with ASD may be more likely to require sedation or general anesthesia (GA) due to their behaviour or the extent and severity of their disease [22,23,24]. Thus, the objective of this retrospective study was to compare the oral health status and treatment needs of children with ASD to healthy controls and to explore the differences in treatment outcomes and additional risks associated with GA referrals.

## 2. Materials and Methods

### 2.1. Study Design and Setting

This retrospective study was reviewed and authorized by the Research Ethics Board at the University of Saskatchewan (REB ID # 848). The study protocol was compliant with the Strengthening the Reporting of Observational Studies in Epidemiology (STROBE) guidelines for reporting observational studies [25] and as previously described [26]. The primary locations for data collection included the teaching clinics at the College of Dentistry, University of Saskatchewan, in Saskatoon, Saskatchewan, Canada.

### 2.2. Participants

Clinical charts for patients who were between 6 and 14 years of age and had an examination at the clinics between 1 July and 30 June 2019, were reviewed. Only patients who received a full comprehensive examination, including radiographs, and treatment plan were considered eligible for inclusion. The study population for children with ASD included those who had a documented diagnosis of ASD based on DSM-5 criteria in their dental record. Age- and gender-matched children were then selected and included as controls if there was no reported intellectual, physical, or developmental disabilities, or chronic diseases. Patient identities were concealed through the assignment of a random ID number for each record.

### 2.3. Variables

For consistency, all records were reviewed by the same individual (KD). The definition and recording of all clinical variables were consistent with methodology used for the oral health component of the Canadian Health Measures Survey [27]. The following variables were included: a child’s age; birth date; gender; location of primary residence (remoteness); type of insurance (if applicable); first dental visit; history of dental examinations; oral hygiene (brushing at least once per day); ASD diagnosis; presence of gingivitis; decayed, missing, or filled teeth (DMFT/dmft); total restorations required; total pulpal therapies required; total extractions required; history of dental trauma; malocclusion classification; presence of crowding; presence of bruxism; Frankl behaviour score; and whether the patient was referred for treatment under GA. The Frankl behaviour scale allows for a quick classification of a child’s behaviour in one of four categories: **‘**definitely positive’, ‘positive’, ‘negative’, and ‘definitely negative’ [28]. The history of dental trauma, brushing at least once per day, presence of bruxism, and presence of crowding were recorded as binary variables (recorded as either ‘yes’ or ‘no’ in the patient’s dental record). Referral for GA was also captured as a binary variable (either ‘yes’ or ‘no’).

The remoteness of location of primary dwelling was established by comparing a patient’s postal code to the Postal Code Conversion File (PCCF 2019) from Statistics Canada as described by Subedi et al. [29]. Briefly, the remoteness index (RI) is determined by estimating the distance between a primary address and any population centres in a given area. As described, remoteness is then further divided into 5 categories based on RI score; easily accessible area (RI score < 0.1500); accessible area (RI score 0.1500 to 0.2888); less accessible area (RI score 0.2889 to 0.3898); remote area (RI score 0.3899 to 0.5532); and very remote area (RI score > 0.5532) [29]. The remoteness of residence variable was then dichotomized into two categories: ‘easily accessible/accessible’ and ‘less accessible/remote/very remote’.

### 2.4. Sample Size

The sample size for this retrospective study was calculated to be able to detect an expected mean difference of at least 2.0 for the primary outcome of mean DMFT/dmft index scores between the two groups. A reference DMFT/dmft value of 2.48 with a standard deviation of 6.06 was used as this represents the national mean value for Canadian children in similar age groups [27]. Additionally, an alpha error of 5%, and a beta error of 20% was factored in the estimate. Based on these values, it was estimated a minimum of 145 patients per group was required for this study.

### 2.5. Statistical Methods

Data was first analyzed to determine means, standard deviations, and proportions for each variable. DMFT/dmft scores were used as a standard to assess the burden of caries including a measure of caries experience (DMFT/dmft > 0) and the prevalence of caries (mean DMFT/dmft index scores). Treatment needs are reported as the proportion in each group who required at least one type of each service. Independent t tests and Chi-squared tests were used to test for difference between means and proportions. Statistical significance was set at *p* < 0.05. The risk for dental treatment under GA was determined using a logistic regression model that considered the whole sample population data and included the independent variables for age, gender, insurance status, remoteness of primary residence, brushing at least once per day, DMFT/dmft index score, Frankl behaviour score, and ASD status. All analysis was conducted using STATA 15 software (StataCorp, College Station, TX, USA).

## 3. Results

A total of 600 charts were reviewed for this study—45 of which were excluded as treatment plan data was missing. From this pool, the charts for 173 children with ASD were identified and included in the analysis. From the remaining 382 charts, 173 age- and gender-matched controls were selected. A basic demographic summary of the sample population is shown in Table 1. There was a significantly higher proportion of males (72.3%) when compared to females (27.7%) diagnosed with ASD (*p* = 0.03). With respect to primary location of residence, the majority of both children with ASD and children in the control group lived in an easily accessible, or accessible area (*p* = 0.05). The majority of both groups also had some form of private dental insurance, but more children with ASD (38.7%) had publicly funded insurance when compared to the control group (31.8%). It was also noted that children with ASD brushed less frequently on average and were significantly older than their control counterparts at their first dental visit (*p* = 0.002).

The overall dental treatment needs and oral health status for the study sample are presented in Table 2. Children with ASD were significantly more likely to have gingivitis (78.0%) when compared to controls (65.9%) (*p* = 0.02). With respect to dental caries experience, over 90% of children with ASD had a DMFT/dmft index score > 1, whereas just under 66% of the control group had a DMFT/dmft index score of greater than 1 (*p* = 0.003). The average DMFT/dmft index score for children with ASD was 8.18 ± 1.62, which was significantly higher than the control group (4.93 ± 0.58) (*p* = 0.007). Children with ASD also had significantly more untreated caries (82.1%) when compared to controls (59.0%) (*p* = 0.01), with an average of 8.23 ± 1.12 teeth needing treatment compared to 4.12 ± 0.28 (*p* = 0.02).

Children in the control group were found to present with Class I occlusion (80.9%) more often, when compared to children with ASD (64.7%) (*p* = 0.03). There was no significant difference in crowding between the two sample populations, but significantly more children with ASD reported bruxism. With respect to behaviour, the majority of children with ASD had a Frankl behaviour score as either ‘definitely negative’ (74.57%), whereas most of the control group had a Frankl behaviour score that was ‘definitely positive’ (64.74%) (*p* = 0.02). The proportion of children who required GA was also significantly higher amongst children with ASD (76.3%) than controls (9.8%) (*p* = 0.002).

A logistic regression model was considered to assess additional risks for GA referrals and the results are presented in Table 3. It was found that within the entire sample population, younger children were significantly more likely to be referred for GA (OR 1.29; 95% CI 1.08*–*1.56) when all other variables were held constant. Children who depended on publicly funded insurance programs were also significantly more likely to be referred for GA (OR 1.63; 95% CI 1.21*–*2.03). Additionally, children who lived in more remote areas (OR 1.90; 95% CI 1.31*–*2.72), had a higher mean DMFT score (OR 1.28; 95% CI 1.09*–*1.47), or had an ASD diagnosis (OR 2.13; 95% CI 1.14*–*2.77) were also significantly more likely to be referred for GA when all other variables were held constant. Gender, brushing frequency and behaviour score were not shown to be significant risk factors associated with dental treatment under GA.

## 4. Discussion

ASD is a complex lifelong developmental disorder that has increased in prevalence over the last 50 years and has become a focus in many fields of research. Knowledge of ASD has grown exponentially, but there have been conflicting findings regarding the severity of oral disease and treatment needs in children diagnosed with ASD [8,12,15,16,17,18,19]. The findings of this research indicate that children with ASD were significantly older at their first dental appointment; brushed less frequently; had higher caries experience and severity; had greater treatment needs; were more likely to have class II occlusion and bruxism; and were more likely to be referred for GA when all other variables were controlled.

This research demonstrates that children with ASD were considerably older than otherwise healthy control children at their first dental appointment. The importance of early dental visits and their relation to disease status is well documented in the literature [30,31]. Oral health related outcomes can be improved at early dental visits aimed at risk assessment and prevention. Visiting a dentist in the early stages of primary tooth development results in fewer treatment requirements, and less indications for use of GA [32,33]. There is an essential need of an early establishment of a dental home, and having earlier dental visits for children with ASD, to reduce the need of extensive treatment under GA [31,33]. Children with ASD in the sample population had a higher DMFT/dmft score than the healthy control children. Higher caries rates have been concurrent with decreased salivary rate or buffer capacity. However, previous research has concluded that children with ASD do not have a significantly lower salivary flow or buffer capacity, and thus the higher DMFT/dmft scores could be a result of decreased brushing frequency, older age at first dental visit, larger time intervals between dental visits, preference for sweet, soft and sticky foods, or increased time of food in the mouth due to food packing [1,2,11]. This is consistent with the present findings as it relates to brushing and the age of first dental visit. The delayed age of visit observed in the sample of children with ASD could be due to caregivers understanding of when to visit the dentist for the first time, anxiety of their child’s anticipated behaviour in the dental chair, barriers to access to care such as cost or transportation, or limited providers who are able to treat children with special needs. Higher DMFT/dmft scores may also be attributed to parents focusing on other health and behavioural issues more than oral health, or lack of education surrounding successful behavioural management strategies available at pediatric dental clinics [34]. Many children with ASD may also require assistance brushing their teeth or more supervision while brushing due to limited manual dexterity, sensory issues, or inability to focus on the task [34]. Children with ASD often require higher levels of attention to the health of the oral cavity, and while oral health is important, it is not always the focus of care for many of these individuals and their caretakers [34]. Similar studies such as Loo et al., have found DMFT scores to be lower in individuals diagnosed with autism however, the sample populations were notably older and therefore the sample demographic allowed us to capture caries in primary dentition to a greater extent [35].

The present results demonstrate that children with ASD were significantly less likely to have a class I occlusion and were significantly more likely to have a class II div I occlusion in comparison to their age and gender matched controls. This finding is consistent with other studies and can be a result of children that have ASD tend to have more parafunctional oral habits such as bruxism, pica, lip biting, tongue thrusting, mouth breathing, or use of a pacifier [36,37,38]. Due to these deviant oral habits, these children may be more prone to having malocclusions [10]. Further research may be required to determine the significance of these factors in causing malocclusions in children with ASD.

This study also confirmed that children diagnosed with ASD were more likely to be referred for treatment under GA. When considering their oral health, children with ASD did present with a greater caries experience, required more extensive treatment, and on average had more difficult behaviour, all indications for the use of GA to complete dental treatment. However, once all these factors were held constant, children with ASD were still more susceptible for GA referrals. Autism is one of the most cited reasons for requesting GA, but the anesthetic is costly and associated with its own health risks [23,24]. The use of GA on children with ASD may be due to variety of reasons such as dental health care providers unwillingness to attempt treatment without the use of GA, or oral aversions of children with ASD. Ultimately, a greater emphasis on prevention is required from both professionals as well as caregivers so as to minimize the need for treatment under GA.

Impaired communication ability, cognitive function, and other psychiatric symptoms may heighten barriers to accessing timely and routine dental care for those with ASD. Children with ASD may not be as cooperative in the dental chair, and in addition may not be able to effectively communicate their pain or oral concerns [29,30]. These challenges further highlight the need for routine dental visits aimed at early diagnosis, prevention, and maintenance. Children with ASD may also have oral aversions that make radiographic assessment difficult leading to improper or delayed diagnoses [39]. A major barrier to access to care for individuals with ASD is finding a dental care provider willing and knowledgeable enough to treat children with developmental disabilities [39]. There are currently a shortage of dentists or specialists that are trained and willing to provide care for these individuals [39]. Poorer oral health may compromise a person’s quality of life due to pain, and may also contribute to systemic illness; therefore, regular and timely dental visits are of utmost importance in populations that already have health deficiencies [39].

This retrospective study design is not without limitations. The records obtained for this study were all from a university teaching center, therefore treatments were completed by various dental providers including faculty, undergraduate students, and residents; therefore, some variability with treatment planning is expected. This study also does not account for the varying degrees of disability with ASD, and oral health status differences may be more apparent with increased severity of ASD. In addition, the demographic profile for patients at the university teaching facilities maybe categorized as high risk their oral health related needs, and therefore might not be truly representative for the population at-large. Data collection was also limited to the information available in the dental records, and there was no opportunity for clarification. Future studies, such as a prospective cohort study would add further insight on this topic.

## 5. Conclusions

Children with ASD may face many barriers in accessing oral health care and according to this study exhibit more unmet needs for dental treatment. This study found that children living with ASD experience poorer oral health, particularly in the primary dentition, and may also be more susceptible to being referred for GA for their treatment. Oral health care professionals should be educated on risk factors associated with ASD and aim to improve prevention, early diagnosis, and management strategies.

## Figures and Tables

**Table 1:** Patient demographic characteristics and oral health behaviour n (%) or mean ± (SD).

Variable	ASD *n* = 173	Control *n* = 173	*p* Value
Age (years)	9.31 ± 1.42	9.31 ± 1.42	*p* = 0.50 ^α^
Gender			*p* = 0.03 ^β^
Male	125 (72.3)	125 (72.3)	
Female	48 (27.7)	48 (27.7)	
Remoteness of primary residence			*p* = 0.05 ^β^
Easily accessible area	55 (31.2)	65 (36.6)	
Accessible area	42 (24.3)	29 (16.8)	
Less accessible area	35 (20.2)	40 (23.1)	
Remote area	31 (17.9)	34 (19.7)	
Very remote area	10 (5.8)	5 (2.9)	
Insurance status			*p* = 0.40 ^β^
Private	97 (56.1)	101 (58.4)	
Public	67 (38.7)	55 (31.8)	
Uninsured	9 (5.2)	17 (9.8)	
Age at first dental visit (years)	5.97 ± 1.18	2.79 ± 1.09	*p* = 0.002 ^α^
Dental visit within the last year	136 (78.6)	143 (82.7)	*p* = 0.39 ^β^
Brushing frequency (>1× per day)	115 (66.5)	152 (88.4)	*p* = 0.02 ^β^

ASD = Autism spectrum disorder; SD = Standard Deviation; DMFT/dmft = Decayed Missing Filled Teeth; Statistical significance *p* < 0.05; ^α^ Independent *t*-test; ^β^ chi-squared test.

**Table 2:** Clinical characteristics of the study population, *n* (%) or mean ± (SD).

Variable	ASD *n* = 173	Control *n* = 173	*p* Value
Gingivitis, *n* (%)	135 (78.0)	114 (65.9)	*p* = 0.02 ^β^
Chronic Periodontitis (pocket depth > 4 mm)	8 (4.6)	11 (6.4)	*p* = 0.58 ^β^
Caries experience (DMFT/dmft > 0)	158 (91.3)	114 (65.9)	*p* = 0.003 ^β^
Caries severity (mean DMFT/dmft)	8.18 ± 1.62	4.93 ± 0.58	*p* = 0.007 ^β^
Untreated caries (>=1)	142 (82.1)	102 (59.0)	*p* = 0.01 ^β^
Mean untreated teeth	8.23 ± 1.12	4.12 ± 0.28	*p* = 0.02 ^α^
Extraction required (>=1)	121 (69.9)	50 (28.9)	*p* = 0.003 ^β^
Mean extractions	3.12 ± 0.57	0.98 ± 0.29	*p* = 0.02 ^α^
Pulpal therapy required, (>=1)	45 (26.0)	23 (13.3)	*p* = 0.02 ^β^
Mean pulpal therapies	2.89 ± 0.41	0.98 ± 0.23	*p* = 0.01 ^α^
History of dental trauma (tooth)	55 (31.8)	18 (10.4)	*p* = 0.03 ^β^
Malocclusion			*p* = 0.03 ^β^
Class I	112 (64.7)	140 (80.9)	
Class II division I	40 (23.1)	13 (7.5)	
Class II division II	15 (8.7)	13 (7.5)	
Class III	6 (3.5)	7 (4.0)	
Crowding	53 (30.6)	48 (27.7)	*p* = 0.39 ^β^
Bruxism	62 (35.8)	18 (10.4)	*p* = 0.003 ^β^
Frankl behaviour score			*p* = 0.02 ^β^
Definitely negative	129 (74.57)	3 (1.73)	
Negative	31 (17.92)	8 (4.63)	
Positive	9 (5.20)	50 (28.90)	
Definitely positive	4 (2.31)	112 (64.74)	
Required GA	132 (76.3)	17 (9.8)	*p* = 0.001 ^β^

ASD = Autism spectrum disorder; GA = General anesthesia SD = Standard Deviation; DMFT/dmft = Decayed Missing Filled Teeth; Statistical significance *p* < 0.05; ^α^ Independent *t*-test; ^β^ chi-squared test.

**Table 3:** Logistic regression model assessing predictors for general anesthesia uses.

Parameter	Odds Ratio	SE	95% CI	*p* Value
Age	1.29	0.12	1.08–1.56	0.02
Gender	1.21	0.39	0.34–2.02	0.32
Public insurance	1.63	0.19	1.21–2.03	0.03
Remoteness of primary residence	1.90	0.41	1.31–2.72	0.02
Brushing frequency > 1× per day	0.71	0.37	0.24–1.54	0.49
Frankl behaviour score Definitely negative (ref) Negative Positive Definitely positive	1.00 0.95 0.84 0.79	0.35 0.45 0.52	0.24–1.86 0.76–1.19 0.63–1.09	0.21 0.09 0.10
Mean DMFT/dmft score	1.28	0.14	1.09–1.47	0.01
ASD Diagnosis	2.13	0.42	1.14–2.77	0.03

ASD = Autism spectrum disorder; SE = Standard error; DMFT/dmft = Decayed Missing Filled Teeth; Statistical significance *p* < 0.05.

## Data Availability

The data presented in this study are available on request from the corresponding author. The data are not publicly available due to privacy.
